# Primary Hyperparathyroidism-Induced Brown Tumor of the Mandible: A Diagnostic Dilemma

**DOI:** 10.7759/cureus.77535

**Published:** 2025-01-16

**Authors:** Debasree Boral, Kanad Chaudhuri, Debanti Giri, Rritam Ghosh, Himanshu Upadhyay

**Affiliations:** 1 Oral Medicine and Radiology, Dr. R. Ahmed Dental College and Hospital, Kolkata, IND; 2 Oral and Maxillofacial Surgery, Dental Implant and Maxillofacial Surgery Centre, Etah, IND

**Keywords:** brown tumour, giant cell tumors, hyperparathyroidism, mandible tumour, parathyroid gland adenoma

## Abstract

Hyperparathyroidism (HPT) is caused by an increase in parathyroid hormone (PTH) production by one or more parathyroid glands. Brown tumors are giant cell lesions that occur due to a defect in bone metabolism in patients with primary and secondary HPT. Brown tumors most commonly affect the skeletal bones including maxillofacial bones. A rare case of a brown tumor involving the mandible in a 23-year-old woman is reported here. This case illustrates the importance of a comprehensive investigation for all lesions in the oral and maxillofacial region and emphasizes the need to consider brown tumors associated with primary HPT as a possible differential diagnosis in patients with lytic bone diseases.

## Introduction

Hyperparathyroidism (HPT) is an endocrine disorder in which parathyroid glands secrete elevated levels of parathyroid hormone (PTH), causing disruption in calcium metabolism [1]. HPT can be classified as primary, secondary, or tertiary. Primary HPT (PHPT) occurs due to hyperactive parathyroid glands in parathyroid adenoma or hyperplasia, and less commonly, parathyroid carcinoma [1]. Secondary HPT occurs in patients with renal insufficiency resulting in vitamin D deficiency, malabsorption, or hypercalciuria. Tertiary HPT, in most cases, develops from secondary HPT and emerges into a more severe condition, with autonomous PTH secretion [1].

Increasing levels of PTH result in osteitis fibrosa cystica (OFC), a skeletal deformity characterized by bony cysts, subperiosteal bone resorption, and long bones with brown tumors [2]. Brown tumor is a benign lytic bone lesion, first described by Henry Jaffe in 1942 [1]. The name “brown tumor” derives from the color, which occurs due to vascularity, hemorrhage, and deposits of hemosiderin [3]. There is a 0.1% prevalence rate for brown tumors, and it tends to be more prevalent among older individuals, with a male-to-female ratio of 1:3 [4,5]. Clinical manifestations of PHPT are related to hypercalcemia, which includes renal calculi, peptic ulceration, cognitive impairment, and bone and joint tenderness. Oral manifestations include mobility, pathologic migration, and premature exfoliation of teeth. Bone involvement is generally considered a late symptom of PHPT. PHPT causes calcium to be released from bones over time. This process can lead to osteopenia and an increased risk of fractures. Localized bone loss can lead to lytic lesions in bone known as brown tumors. It is important to note that such a presentation is extremely rare due to the fact that most cases of PHPT are detected early before symptomatic bone lesions develop because of new blood screening techniques that have been developed [3].

## Case presentation

An endocrinologist referred a 23-year-old female patient to our institution for gradual swelling of the left side of her face for four months. The patient was diagnosed with parathyroid adenoma. Her recent biochemical reports revealed an elevated PTH, serum calcium, and low vitamin D. The combination of hypercalcemia and elevated PTH was diagnostic of PHPT due to a parathyroid adenoma. Laboratory results are shown in Table 1.

**Table 1:** Laboratory findings

Test	Results	Normal range
Serum parathyroid hormone	176 pg/mL	15-65 pg/mL
Serum calcium	12.5 mg/dL	8.6-10 mg/dL
Serum phosphorus	4.51 mg/dL	2.6-4.5 mg/dL
25-hydroxyl vitamin D	21.70 nmol/L	75-250 nmol/L
Alkaline phosphatase	111 U/L	30-120 U/L
24-hour urine calcium	179.40 mg/day	100-300 mg/day
Phosphorous	552 mg/day	400-1300 mg/day
Creatinine level	65 μmol/L	64-104 μmol/L

**Figure 1:**
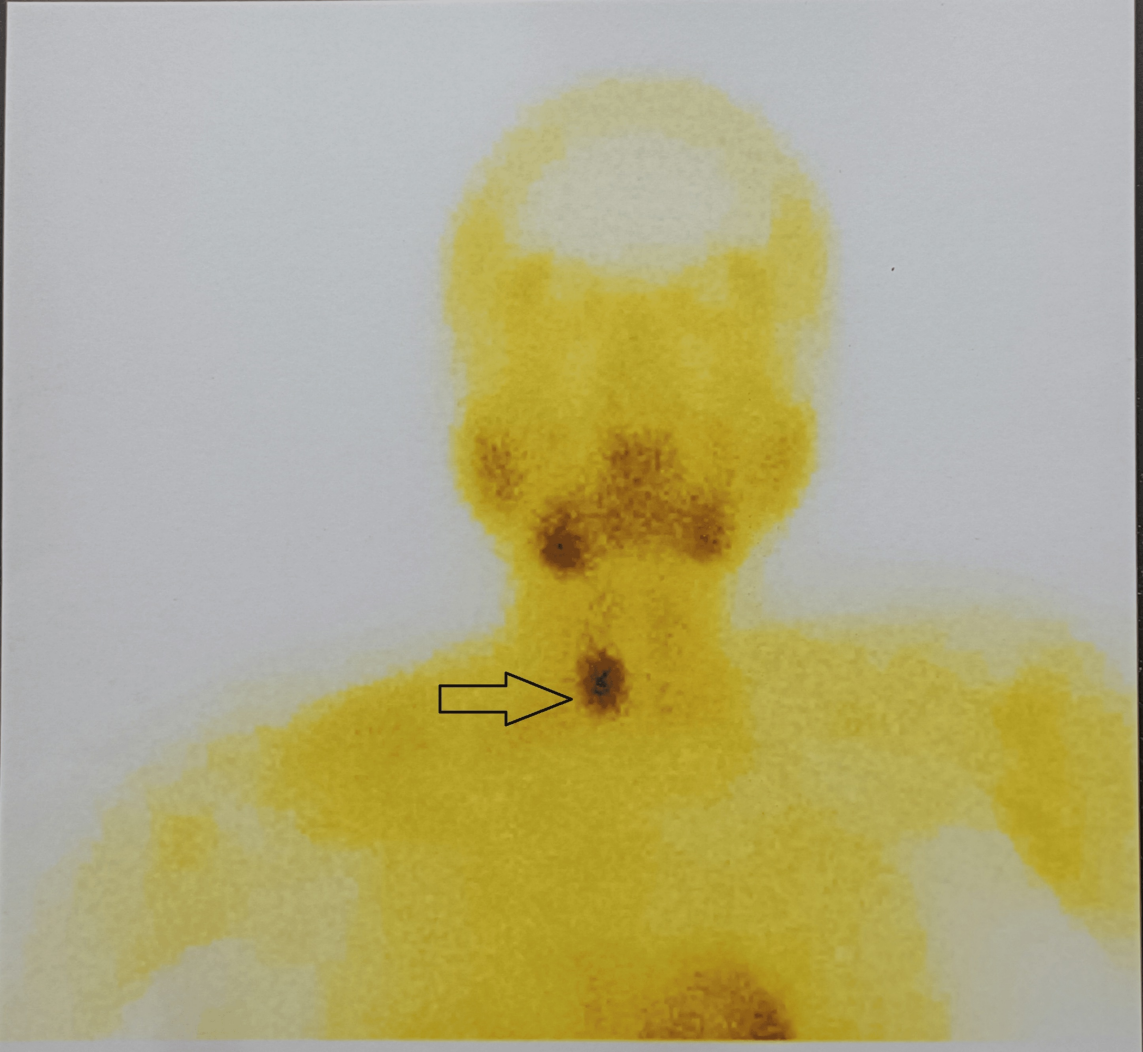
Parathyroid technetium scintigraphy shows abnormally increased tracer uptake in the lower half of the right thyroid bed (black arrow).

Ultrasonography of the neck identified a right parathyroid adenoma. Parathyroid scintigraphy, performed following the intravenous administration of approximately 20 mCi of 99mTc-Sestamibi, revealed a focal nodular area of increased tracer uptake on the delayed static image in the lower half of the right thyroid bed. This finding was interpreted as consistent with a parathyroid adenoma (Figure 1).

**Figure 2:**
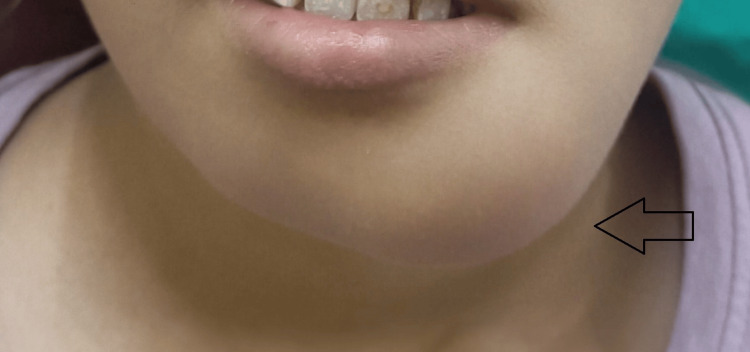
A diffuse swelling was present on the left side of the mandible (black arrow).

There was a history of generalized weakness, lethargy, and weight loss among the patient's symptoms in the past few months. The family history and medical history of the patient were not relevant. On extra-oral examination, a diffuse swelling was noticed on the left lower third of the face, measuring approximately 3 cm × 4 cm, extending superior-inferiorly from the corner of the mouth to 1 cm below the lower border of the mandible, and anteroposteriorly from the midline to 2 cm lateral to the corner of the mouth (Figure 2). The skin over the swelling was shiny and stretched. There was no ulceration, erythema or sinus opening noticed over the swelling. On palpation, the swelling was firm to hard and slightly tender. The temperature of the overlying skin was normal. Left submandibular lymph nodes were palpable, enlarged, tender, and firm in consistency.

**Figure 3:**
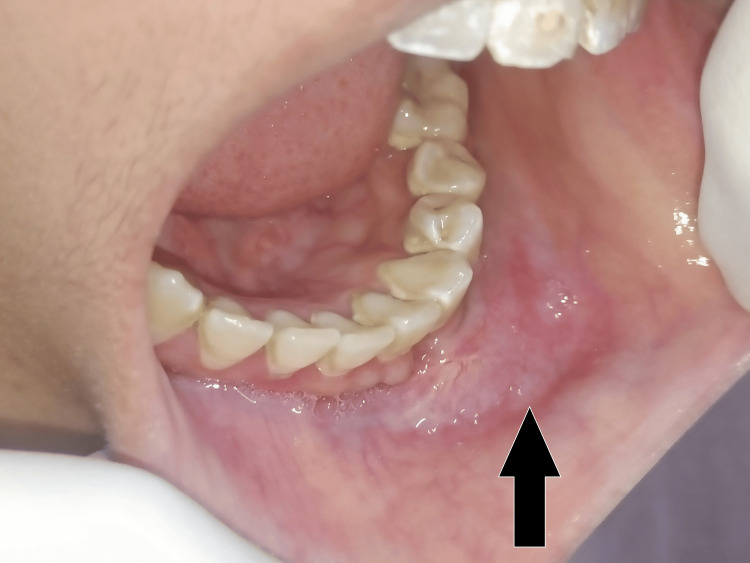
Intraorally, buccal vestibular obliteration was noted (black arrow).

On intraoral examination, buccal vestibular obliteration was noted on the left side of the mandible, extending from the mesial aspect of 31 to the distal aspect of 34 (Figure 3). The mucosa over the swelling appeared blanched and corrugated. The lingual aspect of the mandible was normal. Displacement of teeth and mobility was not found. On palpation, the swelling was firm in consistency and tender, without any discharge or bleeding. All teeth were vital in the affected region.

**Figure 4:**
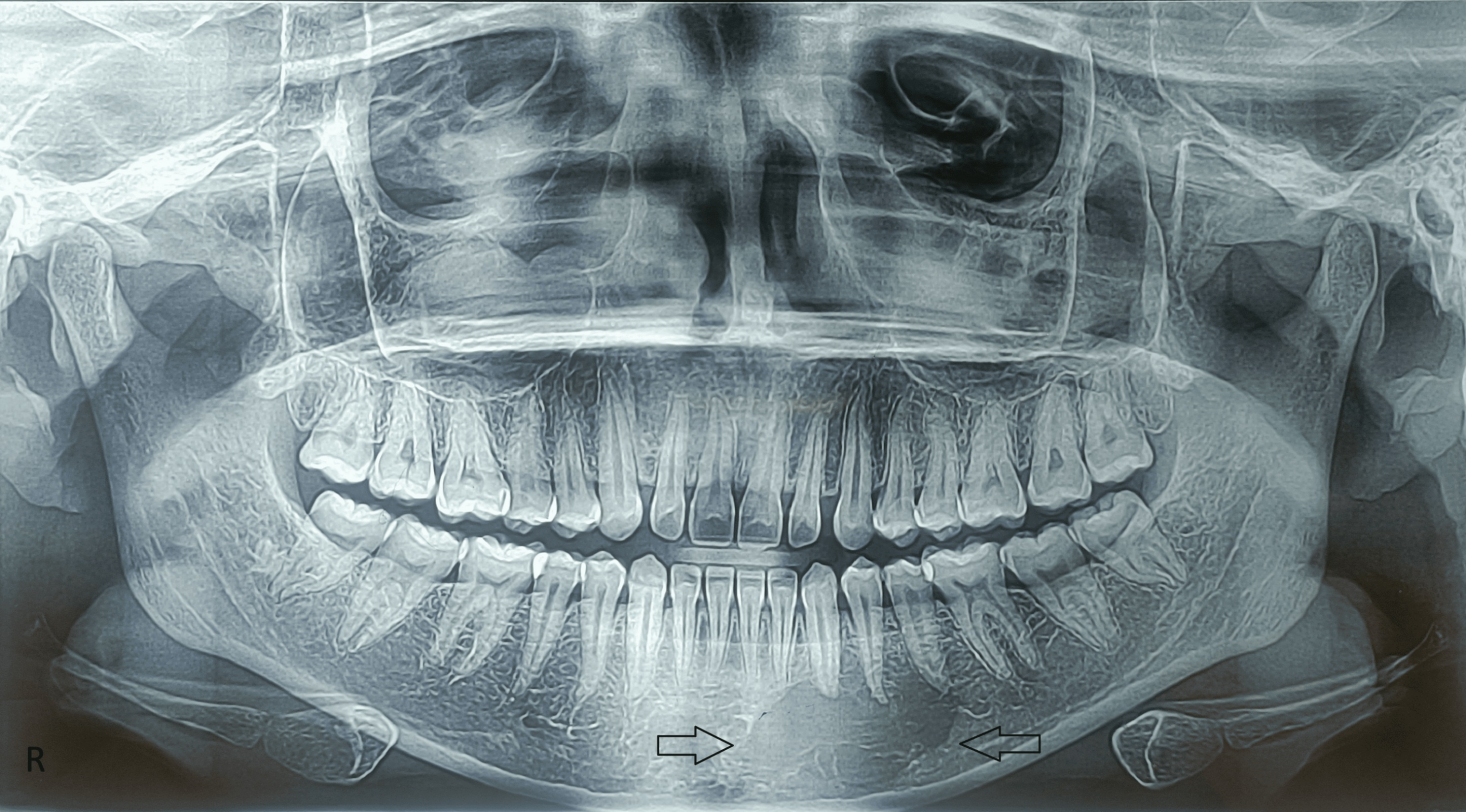
The orthopantomogram shows a large unilocular radiolucency on the left side of the mandible, extending from the mandibular central incisor to the mandibular second premolar (black arrow).

**Figure 5:**
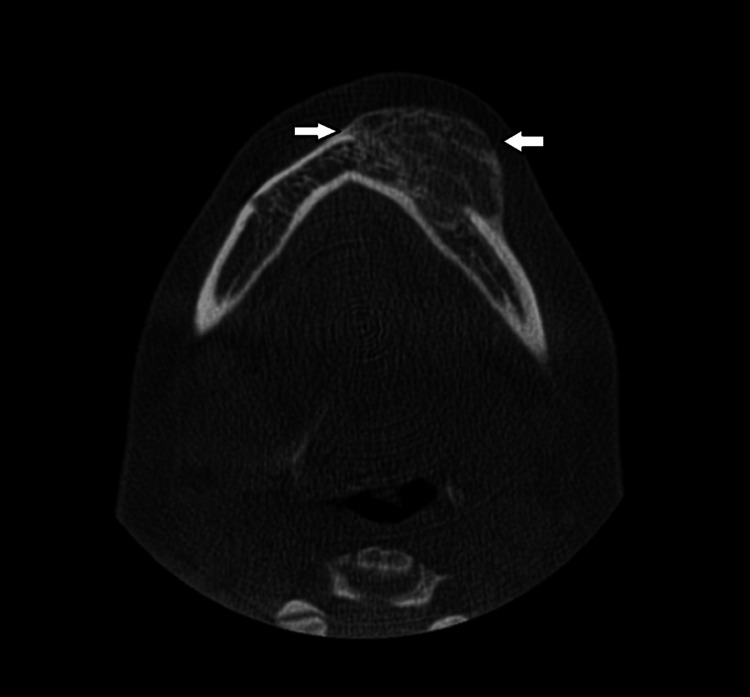
The CBCT scan revealed an expansile, mixed hypodense-hyperdense mass involving the alveolar process of the left body of the mandible, with destruction of the buccal cortical plate (white arrows). CBCT: Cone-beam computed tomography

A provisional diagnosis of brown tumor involving the mandible associated with PHPT and a differential diagnosis of central giant cell granuloma and ameloblastoma were made. The orthopantomogram showed a large unilocular radiolucency on the left side of the mandible, extending from the mesial aspect of 31 to the distal aspect of 35 (Figure 4). Loss of lamina dura was noted for 33, 34, and 35. A cone-beam computed tomography (CBCT) of the patient revealed an expansile, mixed hypodense-hyperdense mass involving the alveolar process of the body of the mandible, extending into adjoining buccal mucosa with thinning and destruction of the buccal cortical plate (Figure 5).

**Figure 6:**
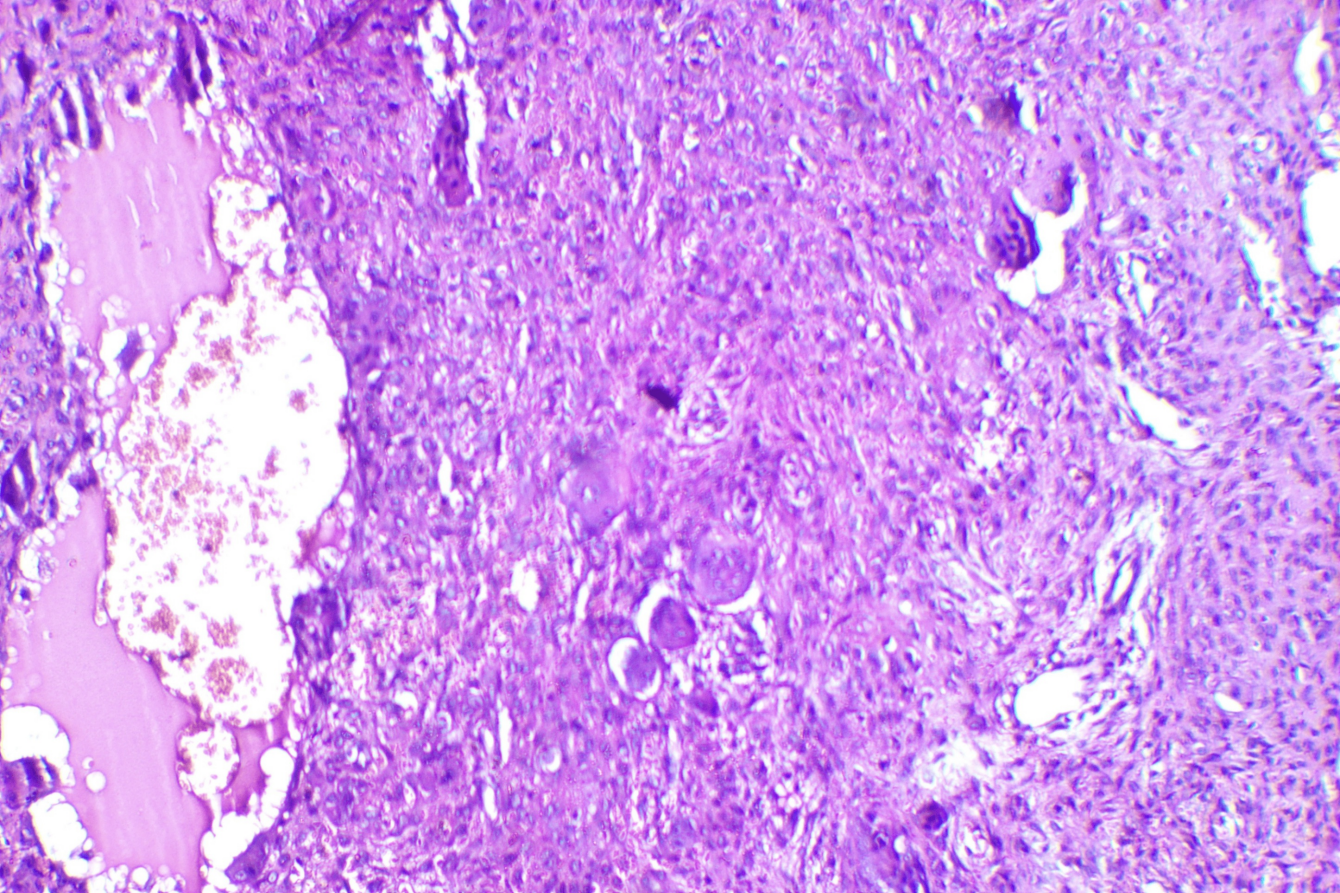
Histological image of brown tumor showing the presence of multiple multinucleated giant cells (H&E stain, X40).

An incisional biopsy of the mandibular lesion was done which revealed a fibrovascular stroma showing non-specific inflammatory infiltrate. The presence of multiple multinucleated giant cells was noted with areas of reactive bone formation and hemorrhage (Figure 6). Hypercellularity noted focally in the stroma, with endothelial cell proliferation was consistent with the diagnosis of a giant cell lesion.

The parathyroid mass was surgically removed under general anesthesia. The patient's recovery was uneventful and she was discharged after one week. Histopathological examination confirmed the diagnosis of parathyroid adenoma. The bony lesion was surgically removed by curettage. A significant reduction in PTH and serum calcium levels was observed after one month, so the patient was given oral calcium supplements in addition to vitamin D3. Within six months, a significant reduction in the size of the oral lesion was observed. Periodic investigations of serum PTH and calcium levels were normal. On telephonic follow-ups after nine and 12 months, the patient reported remarkable improvement without significant residual symptoms.

## Discussion

Brown tumor, also known as OFC generalisata, or Von Recklinghausen’s disease of bone, is a metabolic bone disease that develops in primary, secondary, or tertiary HPT [2]. Since it is a reparative granuloma rather than a neoplastic process, it should be differentiated from other true giant cells of bone [2]. The most common locations for brown tumors are the ribs, the clavicle, and the pelvis [3]. There is a very low incidence of jaw involvement, with the mandible affected more commonly than the maxilla [6,7].

Generally, females are affected between the ages of 50 and 60 due to the hypersecretion of PTH by a parathyroid adenoma [8]. There are a variety of clinical manifestations of these tumors, which can include weakness, weight loss, bone pain, or pathological fractures, as well as progressive swelling of the bones or urinary stones [9]. In the present case, the patient was a 23-year-old female and exhibited none of the classical symptoms of hypercalcemia.

A brown tumor can present clinically as a painless swelling in the jaw or as an exophytic swelling in the jaw. Radiographically, it presents as a well-defined unilocular or multilocular radiolucency, rarely associated with loss of the lamina dura and root resorption of teeth [3].

It can be challenging to differentiate brown tumors from giant cell tumors or other lytic bone lesions due to their clinical and radiographic similarity. Misdiagnosis can result in unnecessary surgery or delaying treatment for HPT. Clinical presentation, laboratory findings, and imaging results must be carefully evaluated by clinicians to differentiate brown tumors from giant cell tumors. PHPT is diagnosed by establishing elevated serum calcium and PTH levels, which are usually elevated [2].

Parathyroid technetium scintiscan is a preferred imaging modality for identifying diseased parathyroid glands before surgical intervention [3]. X-ray absorptiometry (DXA) scans also provide valuable information regarding bone density and brown tumor characteristics [2]. Histologically, brown tumors are non-specific showing a classical population of mononuclear stromal cells mixed with multinucleated giant cells, among which hemorrhagic infiltrates and hemosiderin deposits are often found [10,11]. When a lytic bone tumor is found, a differential diagnosis must be made between brown tumor, central giant cell granuloma, and giant cell tumor of the bone [12].

As a first step in the treatment of primary HPT, a partial parathyroidectomy is performed to control HPT and provoke spontaneous regression of small osteolytic jaw lesions. However, in case of a large symptomatic lesion, surgical excision may be indicated after parathyroid surgery [12].

## Conclusions

Brown tumor is a rare bony disorder as a result of HPT and is frequently misdiagnosed as a giant cell neoplasm. Moreover, estimation of serum calcium and PTH levels is necessary since the radiologic and pathologic makeup of PHPT, giant cell tumors of bone, and central giant cell granulomas resemble each other. In addition, dentists should be familiar with the oral manifestations of systemic diseases and should consider brown tumors as an important differential diagnosis while evaluating patients with osteolytic bone lesions to avoid unnecessary and harmful interventions.
